# Melanoma-derived soluble mediators modulate neutrophil biological properties and the release of neutrophil extracellular traps

**DOI:** 10.1007/s00262-023-03493-5

**Published:** 2023-07-31

**Authors:** Luca Modestino, Leonardo Cristinziano, Marialuisa Trocchia, Annagioia Ventrici, Mariaelena Capone, Gabriele Madonna, Stefania Loffredo, Anne Lise Ferrara, Marilena Romanelli, Ester Simeone, Gilda Varricchi, Francesca Wanda Rossi, Amato de Paulis, Gianni Marone, Paolo Antonio Ascierto, Maria Rosaria Galdiero

**Affiliations:** 1 Department of Translational Medical Sciences (DiSMeT), University of Naples Federico II, 80131 Naples, Italy; 2 WAO Center of Excellence, University of Naples Federico II, 80131 Naples, Italy; 3 Melanoma, Cancer Immunotherapy, and Development Therapeutics Unit, Istituto Nazionale Tumori IRCCS Fondazione “G. Pascale”, 80131 Naples, Italy; 4 Center for Basic and Clinical Immunology Research (CISI), University of Naples Federico II, 80131 Naples, Italy; 5 Institute of Experimental Endocrinology and Oncology (IEOS), National Research Council (CNR), 80131 Naples, Italy

**Keywords:** Neutrophils, Melanoma, Tumor-associated neutrophil, Neutrophil extracellular traps

## Abstract

Polymorphonuclear neutrophils (PMNs) are the main effector cells in the inflammatory response. The significance of PMN infiltration in the tumor microenvironment remains unclear. Metastatic melanoma is the most lethal skin cancer with an increasing incidence over the last few decades. This study aimed to investigate the role of PMNs and their related mediators in human melanoma. Highly purified human PMNs from healthy donors were stimulated in vitro with conditioned media (CM) derived from the melanoma cell lines SKMEL28 and A375 (melanoma CM), and primary melanocytes as controls. PMN biological properties (chemotaxis, survival, activation, cell tracking, morphology and NET release) were evaluated. We found that the A375 cell line produced soluble factors that promoted PMN chemotaxis, survival, activation and modification of morphological changes and kinetic properties. Furthermore, in both melanoma cell lines CM induced chemotaxis, activation and release of neutrophil extracellular traps (NETs) from PMNs. In contrast, the primary melanocyte CM did not modify the biological behavior of PMNs. In addition, serum levels of myeloperoxidase, matrix metalloprotease-9, CXCL8/IL-8, granulocyte and monocyte colony-stimulating factor and NETs were significantly increased in patients with advanced melanoma compared to healthy controls. Melanoma cell lines produce soluble factors able to “educate” PMNs toward an activated functional state. Patients with metastatic melanoma display increased circulating levels of neutrophil-related mediators and NETs. Further investigations are needed to better understand the role of these “tumor-educated neutrophils” in modifying melanoma cell behavior.

**Supplementary Information:**

The online version contains supplementary material available at 10.1007/s00262-023-03493-5.

## Introduction

Melanoma of the skin is the second most prevalent cancer type in males and the fourth in women in the USA. In Europe, skin melanoma is the sixth most frequently occurring cancer (after breast, colorectal, prostate, lung and bladder cancers) and one of the 20 most frequent causes of cancer death [1, 2]. Until recently, the prognosis of metastatic melanoma patients was poor, with a 5-year overall survival (OS) rate lower than 10%. In recent years, the survival of these patients has increased owing to the introduction of novel therapeutic tools, such as BRAF/MEK inhibitors and immunotherapy [3, 4]. Indeed, the 3-year survival rate increased from 22% in patients diagnosed between 2010 and 2012 to 34% in those diagnosed between 2015 and 2017 [1]. A 7.5-year follow-up study demonstrated an OS of 48% for advanced melanoma under combination therapy (nivolumab + ipilimumab) [3]. However, approximately 50% of metastatic melanoma patients still fail to respond or progress after initial therapy [3]. Therefore, there is great interest in finding biomarkers useful in identifying subgroups of patients who will benefit from immunotherapy. Expression of PD-L1 on tumor cells has revealed limitations due to technical issues, sample availability and reproducibility [5]. Analysis of peripheral blood immune cells, which is minimally invasive and repeatable, is a more feasible approach. PD-L1 is expressed on several immune cells, including macrophages and dendritic cells [6, 7]. PD-L1 is also expressed on neutrophils and is associated with several diseases [8–10]. We recently demonstrated that peripheral blood PD-L1^+^ PMN frequency predicts patient prognosis and response to the anti-PD-1 agent nivolumab in patients with stage IV BRAF wild-type melanoma [11].

Increasing evidence highlights the role of polymorphonuclear neutrophils (PMNs) in inflammatory responses in different types of cancer [12, 13], correlating with the clinical outcomes of patients [14, 15]. Ultraviolet irradiation induces PMN chemotaxis in the skin due to the release of High Mobility Group Box 1 (HMGB1) from ultraviolet-damaged keratinocytes. The consequent neutrophilic inflammation promotes angiotropism and distant metastasis of melanoma cells [16]. PMNs exert their effector functions by releasing a plethora of preformed granular enzymes packed in their readily mobilizable granule subsets [13]. They are equipped with three unique types of granules subsets namely primary (azurophilic) granules, secondary (specific) granules and tertiary (gelatinase) granules. Granular enzymes can take part into different phases of malignant initiation and progression and can also contribute to immunosuppression of T cell proliferation by extracellular L-arginine depletion [17–19].

Activated PMNs release an extracellular fibrillary network, termed neutrophil extracellular traps (NETs), composed of nuclear elements (DNA and histones) and granule proteins [20]. NETs are central elements in several pathological conditions, including cancer [20, 21]. In a mouse melanoma regression model, immunotherapy with immune checkpoint inhibitors (ICIs) (e.g., anti-CTLA-4, anti-PD-1 or their combination) increased intratumor neutrophil counts and induced NET-like formation [22]. In humans, melanoma biopsies revealed PMN-releasing NETs in ulcerated melanomas. Interestingly, NETs inhibited the in vitro migration of melanoma cells and exert cytotoxic effects on melanoma cells [23].

To go deeper inside the mechanisms of the interaction between melanoma cells and PMNs, we analyzed the functional characteristics of “tumor-educated” PMNs in melanoma by the use of an in vitro model [15] and evaluated the presence of soluble neutrophil-related mediators in peripheral blood derived from advanced melanoma patients.

## Materials and methods

### Cell cultures and preparation of tumor-conditioned media

Human melanoma cell lines SKMEL28, A375 were obtained from ATCC, cultured and maintained in RPMI 1640 supplemented with 10% of heat-inactivated fetal calf serum (FCS) (endotoxin level < 0.1 EU/ml), 50 U/ml penicillin/streptomycin and 2 mM L-glutamine (Euroclone, Milan, Italy) at 37 °C in a humidified atmosphere containing 5% of CO_2_ and 95% of air. Adult lightly pigmented human epidermal melanocytes (HEMa-LP) were cultured and maintained according to the manufacturer's instructions (ThermoFisher, Waltham, MA, USA). In tissue culture plates, cells were seeded at a confluence of 10–20%. Fresh serum-free medium was added to the cell culture once the cells reached 85–90% confluence. Conditioned medium (CM) was harvested after 24 h, filtered (0.20 µm pore size filter) and stored at – 20 °C [15]. In all the performed experiments, control medium, melanoma-derived CM as well as the medium in which neutrophils were cultured, was supplemented with 5% FCS. Mycoplasma was routinely screened in all cell lines by PCR (Merk, Darmstadt, Germany).

### Neutrophil purification and culture

The Ethics Committee of the University of Naples Federico II approved the study protocol involving the use of human blood cells (Prot. n. 301/18), and the blood donors provided written informed consent in accordance with the principles outlined in the Declaration of Helsinki. Buffy coats from healthy donors (hepatitis B surface antigen, hepatitis C virus and HIV virus negative) were used to isolate granulocytes. Using dextran sedimentation, erythrocytes and leukocytes were separated. PMNs were purified by Ficoll-Paque Histopaque-1077 (Sigma-Aldrich, Milan, Italy) density gradient centrifugation (400×*g* for 30 min at 22 °C) [24]. The EasySep Neutrophil Enrichment Kit (StemCell Technologies, Vancouver, Canada) was used to isolate PMNs from granulocytes (to achieve > 99% purity) [25]. The MACSQuant Analyzer 10 (Miltenyi Biotec, Bergisch Gladbach, Germany) was used for flow cytometry, and FlowJo software version 10 was used to analyze the samples. According to flow cytometric analysis using the anti-CD3, anti-CD14, anti-CD15, anti-CD11b, anti-CD193 (Miltenyi Biotec, Bergisch Gladbach, Germany), anti-CD62L (L-selectin) (BD Biosciences, San Jose, CA, USA) and anti-CD66b (BioLegend, San Diego, CA, USA) antibodies, the cells were > 99% PMNs (Supplementary Figure 1). Doublets, dead cells, debris or eosinophils were excluded from the analysis. The median fluorescence intensity (MFI) or percentage of positive cells was used to express the data.

### Cell migration assay

A 3 µm cell culture insert in 96-well companion plates was used to measure the migration of PMNs toward melanoma CM (Corning Costar, New York, NY, USA). CM or control medium (235 µL) was placed on the companion plates (lower chamber). The insert was filled with 2.5 × 10^6^ PMNs/ml per 75 µL, and PMNs were allowed to migrate for one hour (37 °C, 5% CO_2_). After incubation, the cells were resuspended in 100 µL of PBS and counted by flow cytometry (MACS Quant Analyzer 10, Miltenyi Biotec, Bergisch Gladbach, Germany). In some experiments, PMNs were pre-incubated with mouse monoclonal anti-CXCR1 and/or anti-CXCR2 blocking antibodies (clone 42705 and 48311, respectively, from R&D Systems, Minneapolis, MN, USA) or the corresponding control isotype and subjected to the migration assay as described. All the experiments were run in triplicate.

### Apoptosis assay

PMNs (2.5 × 10^6^ cells/mL) were cultured in A375 CM or control medium, with or without 10 µg/mL mouse monoclonal anti-GM-CSF blocking antibody (clone 3209, R&D System, Minneapolis, MN, USA) or the corresponding control isotype (R&D System, Minneapolis, MN, USA). PMNs were stained with fluorescein isothiocyanate (FITC)-conjugated annexin V and propidium iodide (PI), according to the manufacturer's instructions (Miltenyi Biotec, Bergisch Gladbach, Germany). A MACS Quant flow cytometer (Miltenyi Biotec, Bergisch Gladbach, Germany) and FlowJo version 10 were used for quantification. Live cells were annexin V-PI double-negative. All the experiments were run in triplicate.

### Flow cytometry

PMNs were stimulated with SKMEL28 CM, A375 CM, HEMa CM or control medium for 90 min (37 °C, 5% CO_2_). To determine the viability of the cells (2.5 × 10^5^), Zombie Violet dye (BioLegend, San Diego, CA, USA) was added. The cells were then stained with phosphate buffered saline (PBS) containing 1% FCS for 20 minutes at + 4 °C. Allophycocyanin (APC)-conjugated anti-CD66b (clone REA306, 1:50), VioBlue-conjugated anti-CD193 (clone REA574, 1:10), PerCP-conjugated anti-CD11b (clone REA713, dilution 1:50) and FITC-conjugated anti-CD62L (clone 145/15, dilution 1:50) were used, all from Miltenyi Biotech (Bergisch Gladbach, Germany). The MACS Quant Analyzer 10 and FlowJo software version 10 were used to analyze the results. Doublets and debris (identified based on forward and side scatter properties), dead cells (identified using the Zombie Violet Fixable Viability Kit) and eosinophils (identified based on the CCR3^+^ exclusion gate) were excluded from the analysis. All the experiments were run in duplicate.

### Fluorescence, time lapse and high-content microscopy

Microscopy experiments were conducted using the Operetta High-Content Imaging System (PerkinElmer, Waltham, MA, USA) as previously described [26]. PMNs were cultured overnight in 96-well black CellCarrier plates (PerkinElmer, Waltham, MA, USA), and every 15 min, digital phase-contrast images of 15 fields were taken (20X objective). The dedicated STAR analysis sequence of PhenoLOGIC (PerkinElmer, Waltham, MA, USA) was used to calculate single-cell morphological results [25]. Calculations of the symmetry properties, threshold compactness, axial properties, radial properties and profile are possible using the STAR method [25, 26]. In another set of experiments, to evaluate NET release, PMNs were seeded in 96-well black CellCarrier plates (PerkinElmer, Waltham, MA, USA) within a SKMEL28, A375, HEMa CM or control medium, in the presence or absence of 0.5 µM of a cell impermeant SYTOX Green Nucleic Acid Stain (ThermoFisher, Waltham, MA, USA) at 37 °C and 5% CO_2_ for up to 60 min. Nuclei were stained with bisbenzimide DNA dye Hoechst 33342 (ThermoFisher, Waltham, MA, USA). During the time window, three fields per well of fluorescence microscopy images were taken (10X objective). The dedicated analysis sequence in PhenoLOGIC (PerkinElmer, Waltham, MA, USA) was used to calculate single-cell results. Nuclei staining was used to identify the cells, and NETing cells were identified by their green, cloudy appearance detected in the FITC channel. In some experiments, monoclonal anti-CXCL8/IL-8 and/or anti-GM-CSF blocking antibodies (clones 6217 and 3209, respectively; R&D System, Minneapolis, MN, USA) or the relative control isotype (R&D System, Minneapolis, MN, USA) were added. The number of NETting cells was calculated using the formula (a/b) × 100 [(a) out of the total (b) had produced NETs]. All the experiments were run in triplicate.

### Patients

At the Istituto Nazionale Tumori—IRCCS—Fondazione "G. Pascale" of Naples, Italy, 27 patients with stage IV melanoma, as defined by the seventh edition of the American Joint Committee on Cancer [26], and 22 healthy donors, sex- and age-matched, were enrolled. Peripheral blood was collected and processed at the time of diagnosis. Serum samples were obtained (400 g, + 4 °C) and stored at − 80 °C until use. The Istituto Nazionale Tumori—IRCCS—Fondazione "G. Pascale" of Naples' local ethics committee approved the study (prot. no. 33/17). The study was conducted in accordance with the Declaration of Helsinki guidelines for good clinical practice, as well as international standards.

### Quantification of soluble mediators in culture supernatants or total protein lysates

Using commercially available ELISA kits, the concentrations of CXCL8/IL-8, CXCL1/Gro-α, CXCL2/Gro-β, granulocyte–macrophage colony-stimulating factor (GM-CSF), matrix metalloproteinase 9 (MMP-9) and myeloperoxidase (MPO) in cell-free CM, total protein lysates (0.1% Triton X-100) and patient sera were determined (R&D Systems, Minneapolis, MN, USA). MMP-9 and MPO levels in total protein lysates were expressed in micrograms of protein per milligram of total protein as determined by Bradford protein assay (Bio-Rad, Hercules, CA, USA). The absorbance of the sample was measured at 450 nm using a microplate reader (Tecan, Grodig, Austria, GmbH). The sensitivity ranged from 31.2 to 2000 pg/mL for CXCL8/IL-8 and CXCL1/Gro-α, 15.6 to 500 pg/mL for CXCL2/Gro-β, 7.80 to 500 pg/mL for GM-CSF, 31.2 to 2000 pg/mL for MMP-9 and 62.5 to 4000 pg/mL for other molecules (MPO). All the experiments were run in duplicate.

### Serum NET detection

Concentrations of DNA fragments (mono- and oligonucleosomes) were measured using a Cell Death Detection ELISA kit (Roche, Basel, Switzerland) in serum from melanoma patients and healthy controls. The concentrations of Citrullinated Histone H3 (CitH3) were measured in serum from melanoma patients and healthy controls using an ELISA kit (Cayman Chemicals, Ann Arbor, MI, USA) that uses a specific monoclonal antibody for histone H3 citrullinated at residues R2, R8 and R17 (clone 11D3). A microplate reader (Tecan, Grodig, Austria, GmbH) was used to determine the mono- and oligonucleosomes and CitH3 sample absorbance, respectively, at 405 nm and 450 nm. The ELISA sensitivity ranged from 0.15 to 10 ng/ml (CitH3). All the experiments were run in duplicate.

### Statistical analysis

Statistical analyses were performed using Prism 8 (GraphPad Software). The results are presented as mean ± SEM of a specified number of experiments. The D'Agostino and Pearson normality tests were used to determine whether the distribution was normal, and when necessary, statistical methods were chosen to fit non-normal distributions. Based on the parametric or nonparametric distribution of continuous variables, the groups were compared using Student's t-test or the Mann–Whitney U test. One-way or two-way ANOVA was used, as described in the figure legends. Spearman’s rank correlation analysis was used to determine the correlation between two variables, which was then reported as the correlation coefficient (*r*). Differences were considered statistically significant if the *p*-value was less than 0.05.

## Results

### Melanoma-derived CM induced PMN chemotaxis

**Fig. 1:**
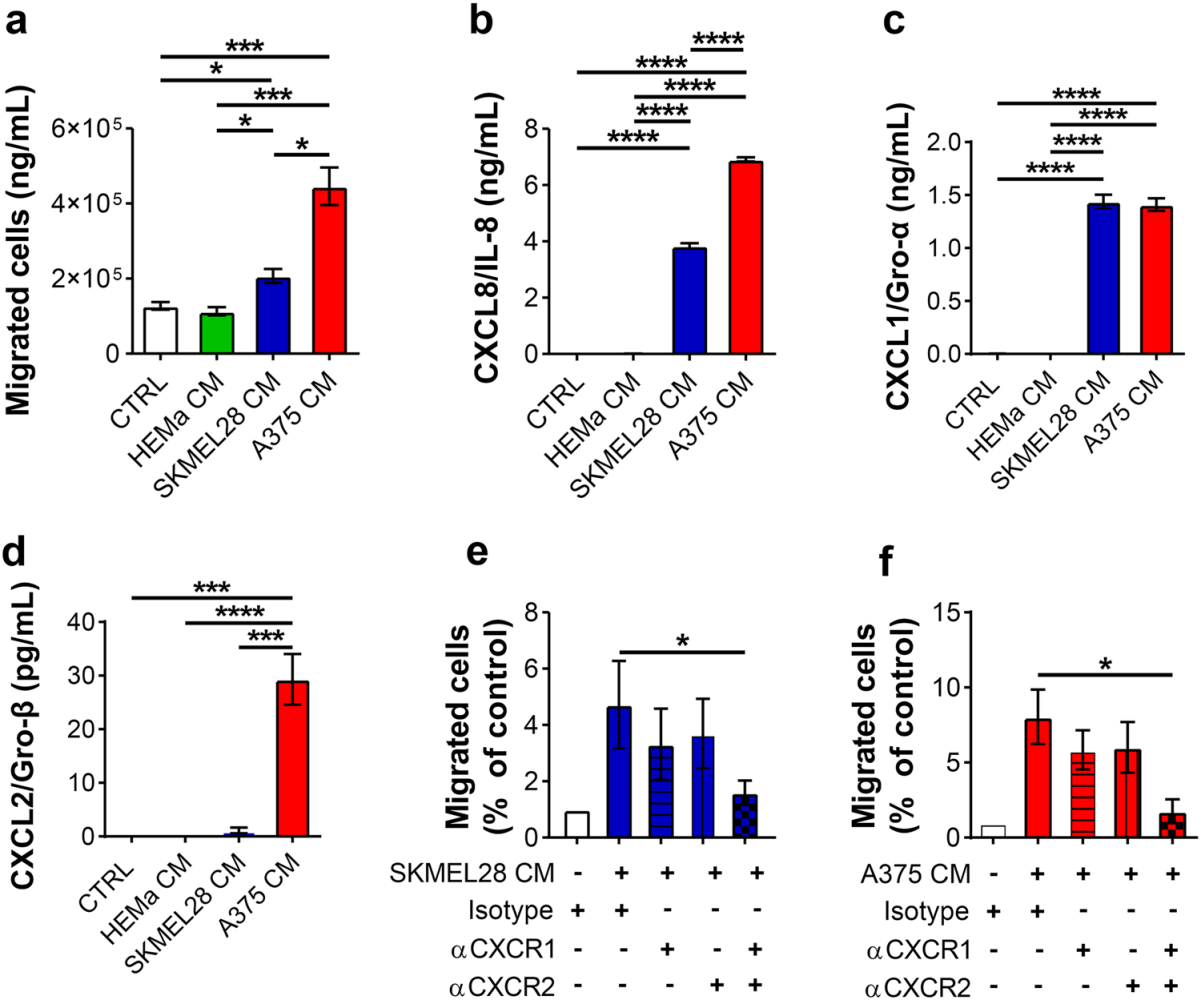
Melanoma-derived conditioned media induced neutrophil chemotaxis. **a** Neutrophil chemotaxis toward melanoma conditioned media (CM) or the control medium (CTRL) was evaluated using 3 µm cell culture inserts in 96-well companion plates. Neutrophils (PMNs) (2.5 × 10^6^ cells/mL per 75 µL) were allowed to migrate (37 °C, 60 min) toward a melanoma CM (SKMEL28 CM, A375 CM), HEMa CM or control medium (235 µL per well). At the end of the incubation, the cells were centrifuged and resuspended in phosphate buffered saline (PBS) (100 µL) and counted by flow cytometry. Data are expressed as migratory cells relative to the control medium (mean ± SEM of five independent experiments). One-way ANOVA and Dunn’s multiple comparison test; *p < 0.05; ***p < 0.005. The CXCL8/IL-8 (**b**), CXCL1/GRO-α (**c**) and CXCL2/GRO-β (**d**) release by HEMa, SKMEL28 and A375 cells was evaluated by ELISA in a conditioned media or in the control medium. Results are expressed as mean ± SEM of five independent experiments; one-way ANOVA and Dunn’s multiple comparison test; ***p < 0.005; ****p < 0.001. Chemotactic activity of neutrophils via a SKMEL28-derived (**e**) or A375-derived (**f**) CM was analyzed in the presence of blocking antibodies directed against CXCR1 and/or CXCR2 (10 µg/mL) or the related isotype control. Migratory PMNs were counted by flow cytometry. The results are expressed as percentage of isotype control (mean ± SEM of ten independent experiments); one-way ANOVA and Dunn’s multiple comparison test; *p < 0.05

In the first group of in vitro experiments, highly purified PMNs (≥ 99%) from peripheral blood of healthy donors were allowed to migrate toward a melanoma CM from SKMEL28 or A375 cell line (SKMEL28 CM, A375 CM), primary melanocyte CM (HEMa CM) or toward control medium. SKMEL28 CM and A375 CM induced PMN chemotaxis as compared to the control medium as well as compared to the HEMa CM (Fig. 1a). In contrast, HEMa CM did not induce the migration of PMNs. These results suggest that SKMEL28 CM and A375 CM selectively released soluble factors capable of inducing PMN chemotaxis. Melanoma cell lines produce several CXC chemokines that can be responsible for PMN chemotaxis [13, 27, 28]. Large quantities of CXCL8/IL-8 and CXCL1/Groα were constitutively released in melanoma CM (~ 8 ng/ml and ~ 2 ng/mL, respectively) (Fig. 1b, c). A375 CM also contained CXCL2/Groβ (0.3 ng/ml) compared to HEMa CM and SKMEL28 CM (Fig. 1d). Blocking CXCR1 and CXCR2 significantly reduced PMN chemotaxis toward SKMEL28 CM (Fig. 1e) or A375 CM (Fig. 1f).

### A375-derived CM promoted neutrophil survival

**Fig. 2:**
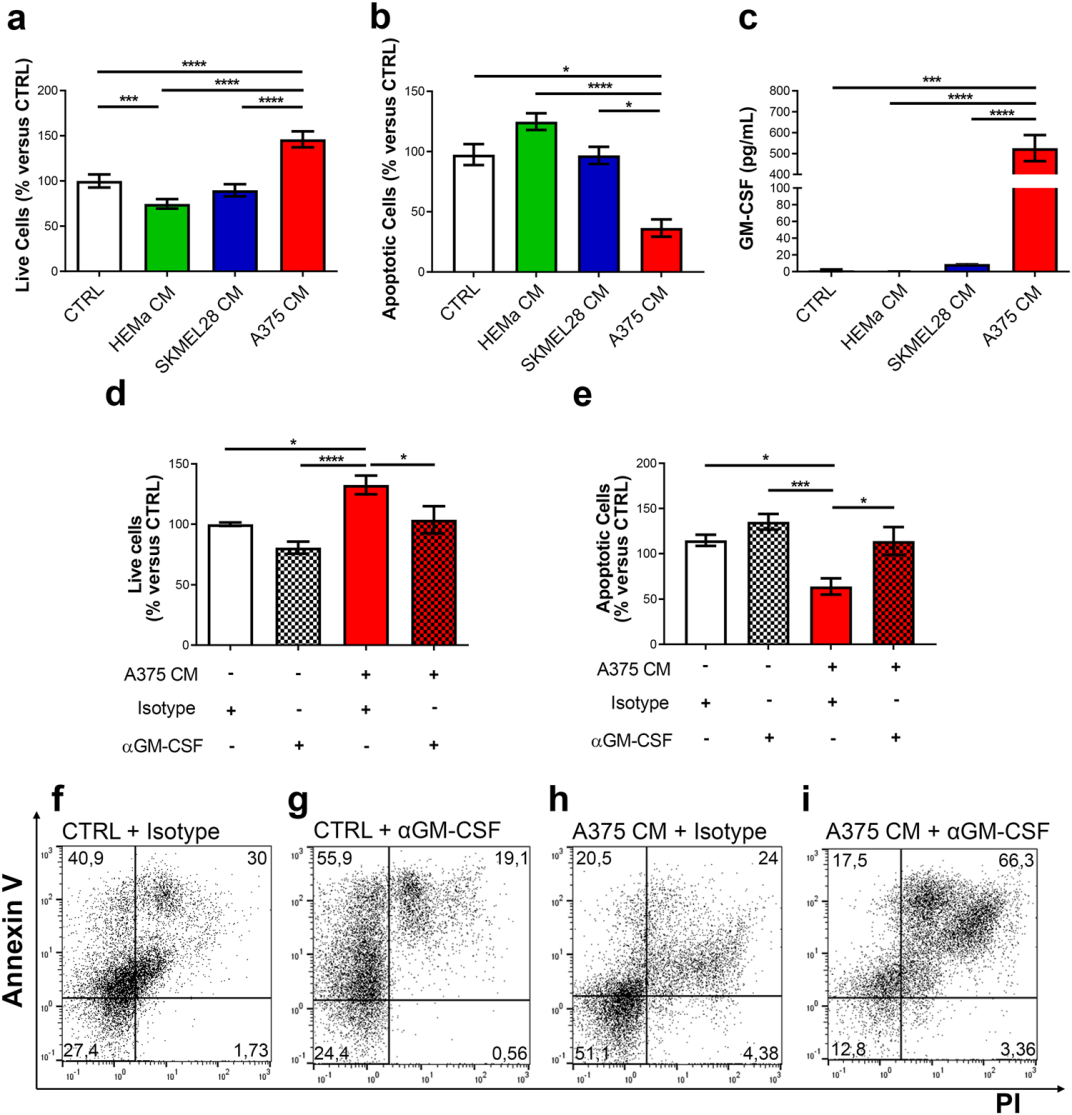
A375-derived CM promoted neutrophil survival. **a**, **b** PMNs were cultured in melanoma CM (SKMEL28 CM, A375 CM), HEMa CM or control medium for 24 h at 37 °C. PMNs were then stained with FITC-conjugated annexin V and propidium iodide (PI) and evaluated by flow cytometry. Results are expressed as percentages of live cells (**a**) and/or apoptotic cells (**b**) compared to control medium (CTRL) (mean ± SEM of five independent experiments); one-way ANOVA and Dunn’s multiple comparison test; *p < 0.05; ***p < 0.005; ****p < 0.001. **c** GM-CSF release by HEMa, SKMEL28 and A375 cells was evaluated by ELISA in conditioned or control media. Results are expressed as the mean ± SEM of five independent experiments; one-way ANOVA and Dunn’s multiple comparison test; ***p < 0.005; ****p < 0.001. **d**, **e** PMN survival in A375-derived CM was evaluated in the presence of an anti-GM-CSF blocking antibody or a relative isotype control (10 µg/mL). At 24 h, cells were stained with FITC-conjugated annexin V and propidium iodide (PI) and analyzed by flow cytometry. Results are expressed as percentages of live cells (**d**) and/or apoptotic cells (**e**) compared to control medium (CTRL) (mean ± SEM of five independent experiments); one-way ANOVA and Dunn’s multiple comparison test. *p < 0.05; ***p < 0.005; ****p < 0.001. **f–i** Representative flow cytometry panels from one of five independent experiments

To investigate whether melanoma-derived soluble factors could modulate PMN lifespan, PMNs from healthy donors were cultured in vitro in SKMEL28 CM, A375 CM, HEMa CM or control medium. After 24 h, PMNs were stained with FITC-conjugated annexin V and PI and subjected to flow cytometry. A375 CM increased the survival and inhibited the apoptosis of PMNs compared with that in the control medium, whereas SKMEL28 CM did not (Fig. 2a, b and Supplementary Table 1). Interestingly, HEMa CM did not increase, but decreased PMN survival compared to the control medium (Fig. 2a). All these data suggest that only the A375 cell line produced soluble factors that increased PMN survival. To dissect the molecular mechanism underlying this anti-apoptotic effect, we evaluated the presence of soluble factors known to increase PMN lifespan in melanoma CM, such as GM-CSF, a key mediator of granulocyte proliferation and differentiation [29]. The A375 cell line constitutively produced high levels of GM-CSF; conversely, the SKMEL28 cell line and HEMa cells did not (Fig. 2c). The pro-survival effect of A375 CM was significantly inhibited by an anti-GM-CSF blocking antibody (Fig. 2d, e and Supplementary Table 1). Figure 2f–i illustrates the representative flow cytometric panels of one out of five independent experiments.

### Melanoma-derived CM induced PMN activation

**Fig. 3:**
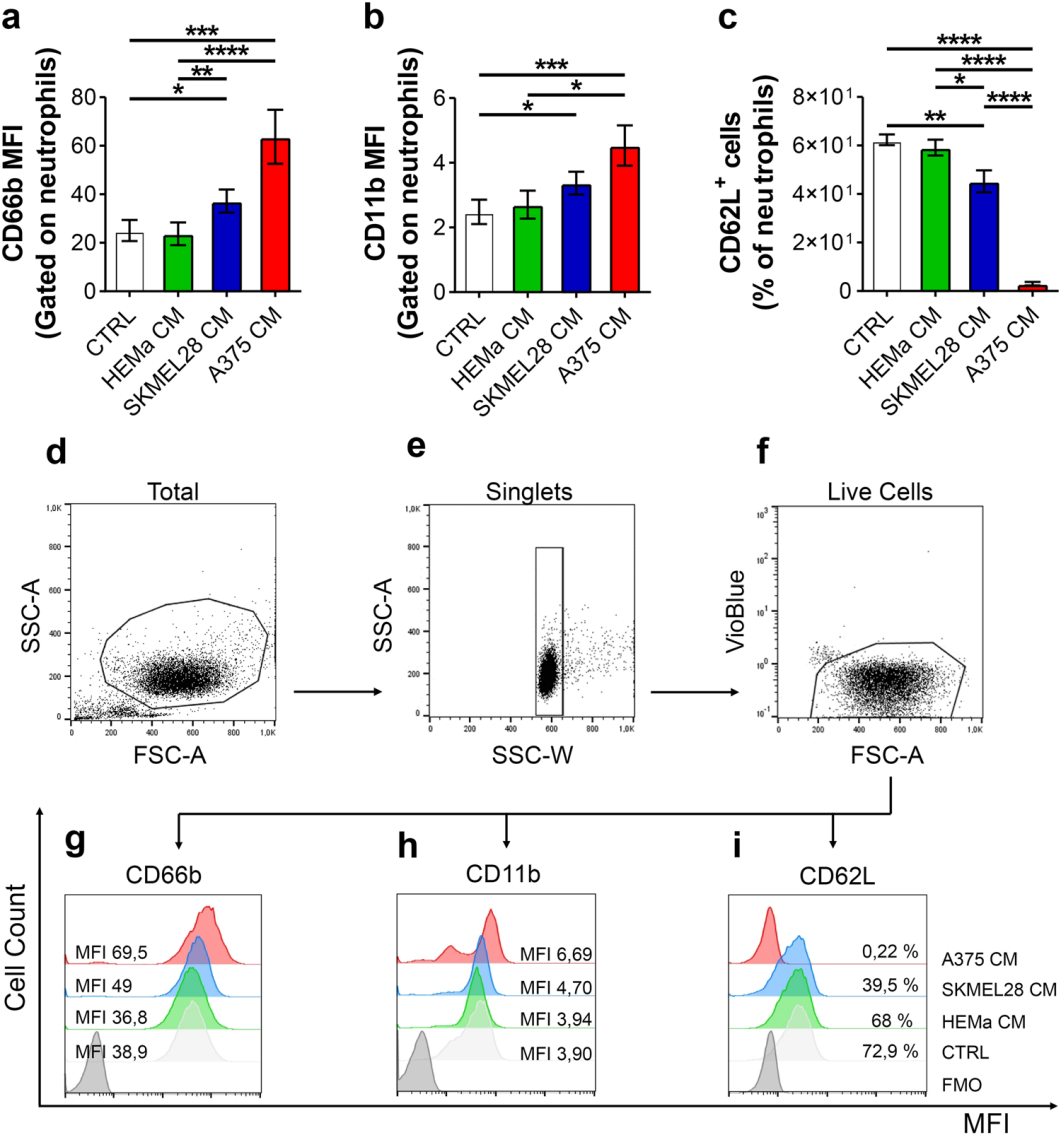
Melanoma-derived CM induced PMN activation. **a**–**c** PMNs were stimulated with melanoma CM (SKMEL28 CM, A375 CM), HEMa CM or control medium for 90 min at 37 °C, stained for neutrophil activation markers CD66b (**a**), CD11b (**b**) and CD62L (**c**) and subjected to cytofluorimetric analysis. The results were expressed as mean fluorescence intensity (MFI) or percentage of positive cells gated on neutrophils (mean ± SEM of seven independent experiments); one-way ANOVA and Dunn’s multiple comparison test; *p < 0.05; **p < 0.01; ***p < 0.005; ****p < 0.001. **d–f** Representative flow cytometric panels were gated on live single cells and show forward (FSC) and side scatter (SSC) of EasySep-purified untouched neutrophils (**d**, **e**). Since Vioblue-positive cells included both dead cells and CCR3^+^ cells (eosinophils), both cells were excluded based on a negative gate (**f**). **g–i** Representative histograms illustrating MFI and cell count for CD66b (**g**), CD11b (**h**) and CD62L (**i**) for one out of seven experiments. *MFI* mean fluorescence intensity, *FMO* fluorescence minus one

PMN activation following melanoma CM, primary melanocyte CM or control medium stimulation was evaluated by flow cytometry. Under basal conditions, PMNs showed minimal expression of CD66b and CD11b, which rapidly increased after incubation with A375 CM compared to the control medium, as well as compared to HEMa CM (Fig. 3a, b, g, h). CD66b expression also increased after incubation with SKMEL28 CM compared with control medium and compared to HEMa CM and a similar trend was also observed for CD11b expression (Fig. 3a, b, g, h). Conversely, under resting conditions, PMNs highly expressed CD62L, which rapidly decreased upon stimulation with A375 CM (Fig. 3c, i). These data indicate that A375 CM, and to a lesser extent SKMEL28 CM, efficiently activated (CD66b and CD11b upregulation, CD62L shedding) human primary PMNs. In contrast, primary melanocyte CM did not induce PMN activation. Figure 3d–i shows representative flow cytometry panels with the specific gating strategy and related histograms.

### A375-derived CM modified neutrophil morphology and kinetic properties

**Fig. 4:**
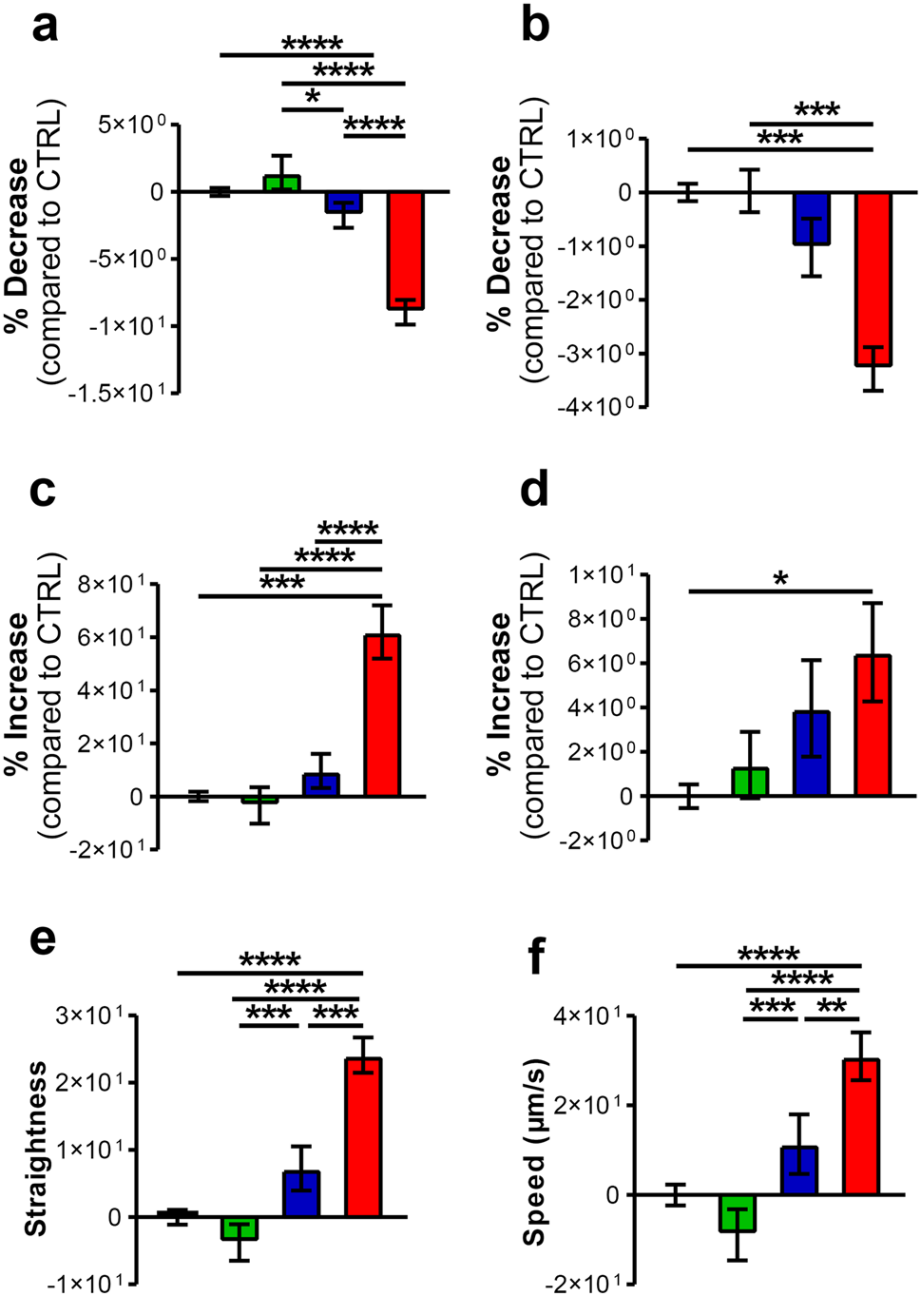
A375 CM induced morphological changes and kinetic properties in PMNs. **a**–**d** PMNs were stimulated with melanoma CM (SKMEL28 CM, A375 CM), HEMa CM or control medium for 16 h at 37 °C and then imaged using an Operetta high-content imaging system at 20 × magnification. The images were analyzed using Harmony software with PhenoLOGIC (PerkinElmer) and a dedicated analysis sequence (morphological properties, method STAR) to evaluate the axial length (**a**), roundness (**b**), asymmetry (**c**) and cell area (**d**). The results are expressed as an increase or decrease compared to the control (mean ± SEM of five independent experiments); *p < 0.05; ***p < 0.005; ****p < 0.001. **e**, **f** In the same time window, digital phase-contrast images of 15 fields/well were captured every 15 min via a ×20 objective in the Operetta high-content imaging system. PhenoLOGIC (PerkinElmer) was employed for image segmentation and to calculate single-cell kinetic properties, straightness (**e**) and speed (**f**). The results are expressed as the mean ± SEM of five independent experiments; one-way ANOVA and Dunn’s multiple comparison test **p < 0.01; ***p < 0.005; ****p < 0.001

Using a live cell imaging system, we measured and tracked changes in morphological characteristics at the single-cell level and quantitatively determined these morphological feature distributions in response to the culture conditions [30]. PMNs treated with A375 CM showed lower axial length (Fig. 4a) and roundness (Fig. 4b), and increased asymmetry (Fig. 4c) and cell area (Fig. 4d). These morphological changes typically occur following PMN stimulation by inflammatory cytokines and growth factors [31]. Moreover, PMNs stimulated with A375 CM showed an increased straightness (Fig. 4e) and speed (Fig. 4f) compared with control medium, as well as compared with HEMa CM and with SKMEL28 CM. Interestingly, SKMEL28 CM and HEMa CM did not modify PMN morphology or kinetic properties, suggesting that only the A375 cell line produced soluble factors capable of modulating these aspects of PMNs.

### Melanoma-derived CM induced MMP-9 and MPO release

**Fig. 5:**
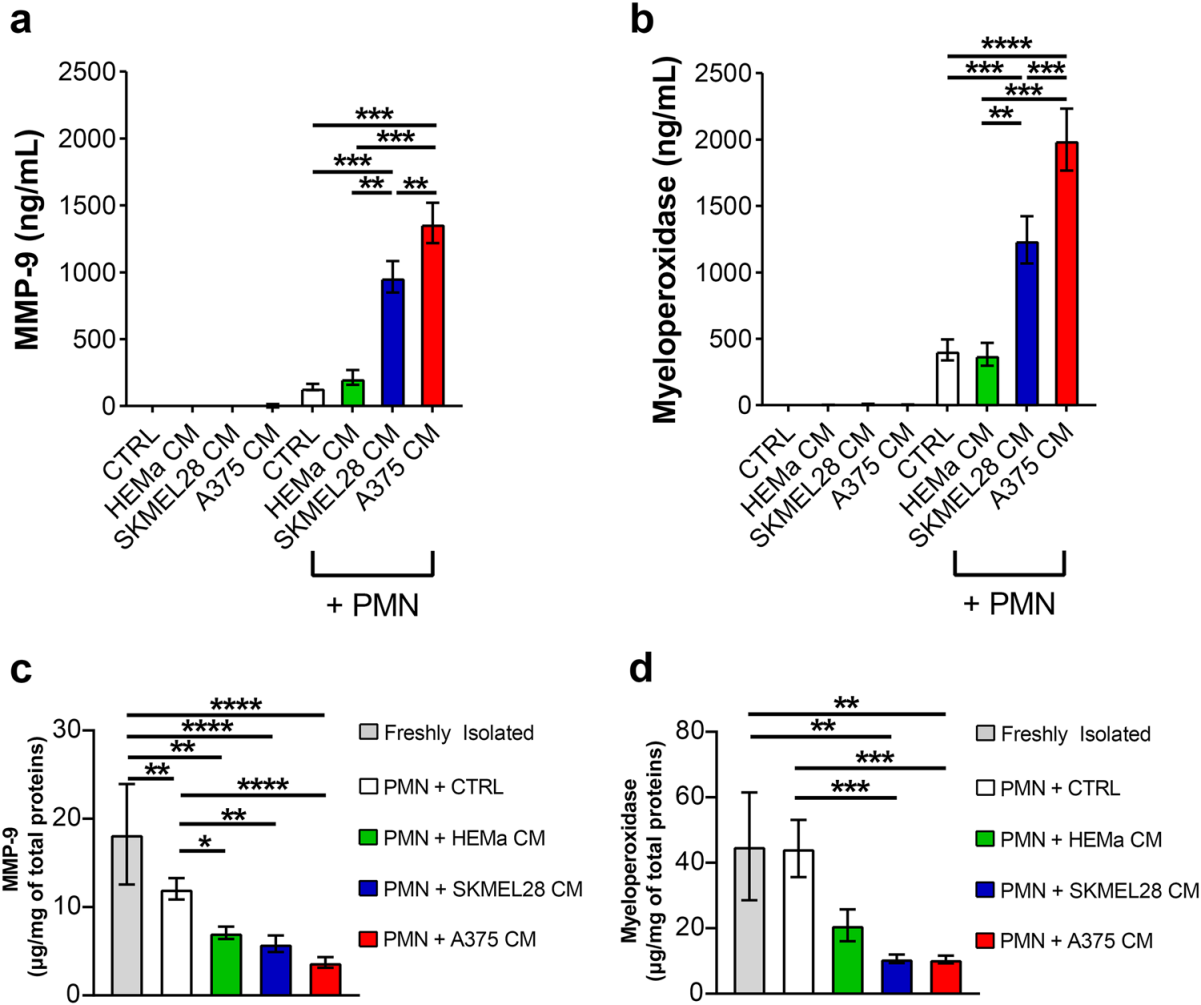
Melanoma-derived CMs induced MMP-9 and MPO release. **a**–**d** PMNs were cultured in melanoma CM (SKMEL28 CM, A375 CM), HEMa CM or control medium for 18 h at 37 °C. At the end of the incubation, PMNs were harvested and centrifuged (600×*g*, 4 °C, 5 min), and the supernatants were collected. The extracellular content of MMP-9 (**a**) and MPO (**b**) from melanoma cell lines and PMNs as well as intracellular concentration of MMP-9 (**c**) and MPO (**d**) in neutrophils after cell lysis (Triton X-100, 0.1%) were evaluated by an ELISA. Intracellular levels of MPO and MMP-9 were also measured in freshly isolated PMNs (**c**, **d**; gray bars). The results were expressed as mean ± SEM of five independent experiments; one-way ANOVA and Dunn’s multiple comparison test; *p < 0.05; **p < 0.01; ***p < 0.005; ****p < 0.001

PMNs were cultured in SKMEL28 CM, A375 CM, HEMa CM or control medium, and the extracellular levels of MMP-9 and MPO in PMN supernatants were measured by ELISA. MMP-9 and MPO concentrations were increased in PMN supernatants upon SKMEL28 CM or A375 CM stimulation compared with PMNs cultured in HEMa CM or control medium (Fig. 5a, b). Accordingly, MMP-9 (Fig. 5c) and MPO (Fig. 5d) intracellular levels were reduced in PMNs following stimulation with SKMEL28 CM or A375 CM compared to PMNs cultured in control medium as well as compared to freshly isolated (non-activated) PMNs. These results suggest that SKMEL28 CM and A375 CM mediate MMP-9 and MPO release from tertiary and primary granules, respectively, in human primary PMNs.

### Melanoma-derived CM induced NET release from human neutrophils

**Fig. 6:**
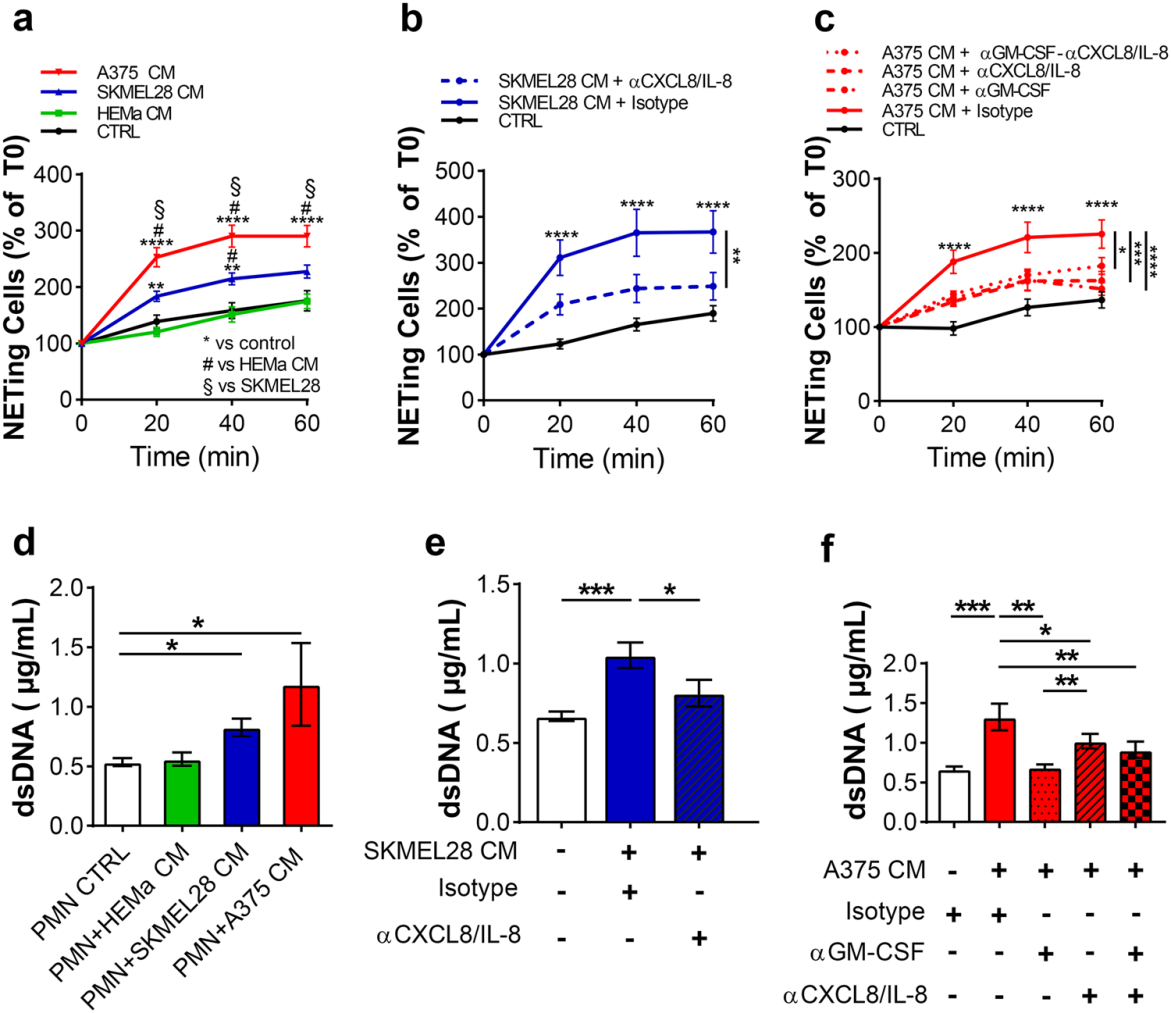
Melanoma CMs induced NET release from human PMNs. **a**–**c** PMNs (1 × 10^6^ cells/mL) were seeded in a 96-well CellCarrier plate and cultured in A375, SKMEL28 or HEMa CM or in a control medium (37 °C, up to 60 min) in the presence or absence of anti-CXCL8/IL-8 and/or anti-GM-CSF blocking antibodies or the relative isotype control (10 µg/mL) in the presence of the cell-impermeant SYTOX Green Nucleic Acid Stain (0.5 µM). The percentage of cells that produced NETs over total cells was calculated. Data are expressed as percentage of NETing cells versus time 0 (mean ± SEM of five independent experiments using five different donor samples). Two-way ANOVA and Bonferroni multiple comparison test (**a**) or Dunnett’s multiple comparison test (**b**, **c**); *p < 0.05; **p < 0.01; ***p < 0.005; ****p < 0.001. **d**–**f** dsDNA levels in supernatants of neutrophils cultured with A375, SKMEL28 or HEMa CM or in control medium (37 °C, 60 min), in the presence or absence of anti-CXCL8/IL-8 and/or anti-GM-CSF blocking antibodies or the relative isotype control (10 µg/mL), were measured by Quant-iT™ PicoGreen™ dsDNA Assay Kit (ThermoFisher). Results were expressed as mean ± SEM of five independent experiments; one-way ANOVA and Dunn’s multiple comparison test; *p < 0.05; **p < 0.01; ***p < 0.005

To evaluate the ability of the melanoma cell lines to induce NET release from PMNs, we used the Operetta High-Content Imaging Screening System. A375 CM and SKMEL28 CM induced NET release from PMNs after ~ 20 min of stimulation (Fig. 6a). Control medium and HEMa CM did not induce NET release from PMNs (Fig. 6a). These results suggest that SKMEL28 CM and A375 CM rapidly and selectively induced NET release from the PMNs. Melanoma cell lines autocrinously produce significant quantities of CXC chemokines and GM-CSF [32]. GM-CSF and CXCL8/IL-8 can induce NET release from PMNs [33, 34]. Anti-CXCL8/IL-8 blocking antibody inhibited SKMEL28 as well as A375 CM induced NET release (Fig. 6b, c) and anti-GM-CSF blocking antibody inhibited A375 CM induced NET release (Fig. 6c). The combination of anti-CXCL8/IL-8 and anti-GM-CSF blocking antibodies inhibited A375 CM induced NET release (Fig. 6c). The Quant-iT™ PicoGreen™ dsDNA assay confirmed the presence of dsDNA, and the results were obtained using the Operetta High-Content Imaging Screening System (Fig. 6d–f).

### Serum levels of NET biomarkers and neutrophil-related mediators in melanoma patients

**Fig. 7:**
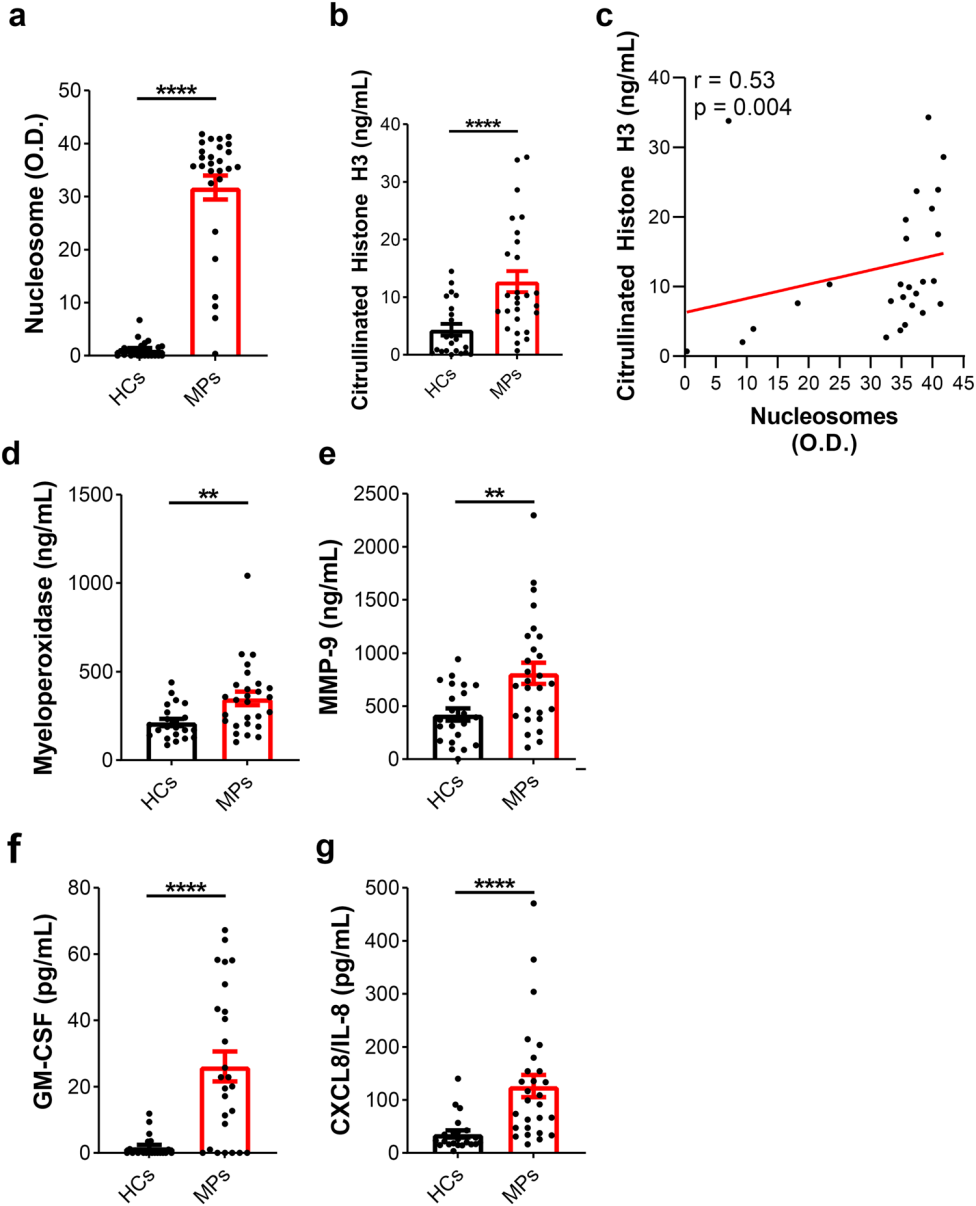
Serum levels of NETs in melanoma patients. Serum concentrations of nucleosomes (**a**) and citrullinated histone H3 (CitH3) (**b**) in melanoma patients (MPs, red borders) and healthy controls (HCs, black borders) were measured by Cell Death Detection ELISA (Roche) and Citrullinated Histone H3 (clone 11D3) ELISA kit (Cayman), respectively. **c** Correlation between serum concentrations of nucleosomes and citrullinated histone H3 (CitH3) in melanoma patients. Spearman correlation test; *r* = 0.53; *p* = 0.0043. Serum concentrations of MPO (**d**), MMP-9 (**e**), GM-CSF (**f**) and CXCL8/IL-8 (**g**) in melanoma patients (MPs, red borders) and healthy controls (HCs, black borders) were measured by ELISA. Results were expressed as mean ± SEM; Student’s *t* test or Mann–Whitney *U* test according to the parametric or nonparametric distribution of the variables. **p < 0.01; ****p < 0.001

Circulating levels of nucleosomes and CitH3 were higher in metastatic melanoma patients than in healthy controls (Fig. 7a-b). Interestingly, circulating levels of nucleosomes and CitH3 were positively correlated (Fig. 7c). We also found that the circulating levels of MPO (Fig. 7d), MMP-9 (Fig. 7e), GM-CSF (Fig. 7f) and CXCL8/IL-8 (Fig. 7g) were higher in metastatic melanoma patients than in healthy controls.

## Discussion

In this study, we describe that soluble factors derived from human melanoma cells influence several biological properties of PMNs relevant to cancer pathology. Melanoma cells recruit PMNs, promote their survival and activation, and modify PMN morphology and kinetic properties. Moreover, melanoma cells upregulate the pro-inflammatory activities of neutrophils as well as the expression of tumor-promoting factors, such as MPO, MMP-9 and NETs. Finally, in a cohort of stage IV melanoma patients, we found increased circulating levels of neutrophil-related mediators, such as MMP-9, MPO, GM-CSF and CXCL8/IL-8, as well as increased levels of NET biomarkers, compared to healthy controls.

Among the innate immune cells involved in melanoma initiation and progression, PMNs have been investigated in only a few studies. In an in vivo melanoma model, ultraviolet (UV) irradiation induced a TLR4/MYD88-driven neutrophilic skin inflammatory response initiated by high mobility group box 1 (HMGB1) release from ultraviolet-damaged keratinocytes. TLR4-driven neutrophilic inflammation promoted distant metastasis [16]. In humans, the circulating neutrophil-to-lymphocyte ratio (NLR) has been used as a prognostic biomarker for melanoma [35, 36], although these findings have some limitations due to PMN heterogeneity [37]. We demonstrated that peripheral blood PD-L1^+^ PMN frequency predicts patient prognosis and response to the anti-PD-1 agent nivolumab in patients with stage IV BRAF wild-type melanoma [11]. To go deeper inside the mechanisms linking melanoma cells and PMNs, we investigated the cross talk between melanoma cell lines and human PMNs purified from peripheral blood of healthy donors. The A375 and SKMEL28 cell lines, two of the most studied human melanoma cells, were chosen as models of different aggressiveness, as demonstrated in vitro and in vivo [38, 39].

In the first series of in vitro experiments, we found that melanoma-derived CM induced PMN chemotaxis in vitro, whereas CM from primary melanocytes did not. Melanoma cells constitutively released CXCL8/IL-8, CXCL1/Groα and CXCL2/Gro-β, and CM induced PMN chemotaxis was dependent on CXCR1/2 activation. Melanoma-derived chemokines act as both autocrine and paracrine mediators [40, 41]. Autocrine loops sustain tumor cell activation, reprogramming and metastasis. Paracrine loops orchestrate tumor behavior through the engagement of receptors expressed by stromal and immune cells. In human melanoma biopsies, *CXCL1/Groα* and *CXCL2/Groβ* genes were over-expressed in metastatic patient samples compared to non-metastatic ones. *CXCL1/Groα* expression independently predicted the occurrence of metastases in a patient cohort [42]. CXCL8/IL-8 autocrinously activates oncogenic signals, pro-metastatic pathways and angiogenesis [43]. Noteworthy, CM derived from primary melanocytes failed to induce PMN chemotaxis, and indeed, primary melanocyte CM did not contain relevant levels of CXCL8/IL-8 or CXCL1/2. These results suggest that chemokine production and the chemotactic effect exerted on PMNs are specific to cancer cells and are related to the acquisition of specific features by melanoma cells during disease progression. Notably, inhibition of both CXCR1 and CXCR2 was necessary to block PMN chemotaxis, highlighting the pleiotropic and redundant potential of melanoma-derived chemokines.

The more aggressive melanoma cell line, A375, produced high levels of GM-CSF and increased PMN survival. In contrast, the less aggressive SKMEL28 cells and primary melanocytes did not produce GM-CSF or modified PMN survival. A375 CM activated PMNs and modified their morphology and kinetic properties. In contrast to the chemotactic activity, the effects on PMN survival and kinetic properties were differentially regulated by the two melanoma cell lines, according to their aggressiveness. Indeed, only the CM derived from the more aggressive A375 cell line increased PMN survival and modified their morphology and kinetic properties, whereas the CM from the less aggressive SKMEL28 cell line and from primary melanocytes did not. These results suggest that beyond PMN recruitment, which could be a “bystander effect” of the production of chemokines by melanoma cells, the modulation of additional PMN biological properties is finely tuned according to melanoma malignancy.

PMNs contain several preformed granular enzymes that can be rapidly released [13]. We demonstrated that melanoma CM, but not primary melanocyte CM, were able to induce the PMN release of MPO and MMP-9, known angiogenic and pro-tumorigenic factors [44]. MPO activates proMMP-8 and proMMP-9 and regulates matrix MMP-7 activity in vitro [45], supports a hypermutagenic environment owing to the action of MPO-derived oxidants [46] and plays important roles in NET formation and ROS production [33]. MMP-9 exerts several functions in different phases of cancer progression [44, 47]. Neutrophil exocytosis of gelatinase and azurophil granules is also important for the activation of Arginase-1, which, in turn inhibits T cell proliferation by depleting extracellular L-arginine [17]. NETs contribute to various aspects of cancer development and metastasis formation [20, 21, 48]. In an experimental melanoma model, NETs within the tumor microenvironment (TME) promoted tumor progression [49]. In contrast, in an in vitro model, NETs decreased melanoma cell viability and migration [23]. In our experimental setup, A375- and SKMEL28-derived CM induced NET formation, whereas primary melanocyte CM and control medium did not. Inhibition of GM-CSF and/or CXCL8/IL-8 inhibited NET formation induced by A375 CM, and blockage of CXCL8/IL-8 inhibited NET induced by SKMEL28 CM. Interestingly, the release of NETs induced by melanoma CM occurred rapidly after approximately 20 min of stimulation. Moreover, we found that PMNs continued to function even after NET formation. These findings support the alternative pathway of NET formation, which is a crucial process that begins minutes after activation and occurs without cell death [33, 50]. The latter form of NET production seems to better fit the role of PMNs in cancer progression, since the preservation of PMN viability allows PMN to exert additional functions in the TME.

To evaluate NET release, we used a high-throughput High-Content Image System that allows uniform standardization of cell image acquisition and analysis using dedicated software [51]. Although the majority of published studies uses automated NET quantification based on microscopy, they are often not fully automated and rely on an increase in staining area as the only criterion for determining the extent of NET formation without knowledge at the single-cell level [52, 53]. To further verify these results, we investigated the in vitro release of dsDNA, which is used as a NET biomarker [54]. Furthermore, serum concentrations of neutrophil-related factors (MPO, MMP-9, CXCL8/IL-8 and GM-CSF) and NET biomarkers (nucleosomes and CitH3) were higher in stage IV metastatic melanoma patients than in healthy controls. Some of these mediators, such as MMP-9 and GM-CSF, are predictors of melanoma progression [55, 56]. However, in vivo*,* NET formation is not always reflected by the quantitative presence of circulating DNA. DNA complexes may develop as a result of cell death caused by neutrophilic inflammation [57]. Therefore, we used two different additional markers (nucleosomes and CitH3) to detect the serum levels of NETs. In particular, CitH3 serum levels, considered a more reliable indicator of NET formation, were measured to corroborate our findings [21, 58]. Collectively, our results demonstrate that melanoma patients display higher levels of neutrophil-related mediators and NETs than healthy controls.

This study has some limitations. Additional experiments are necessary to understand the role of these “melanoma-educated” PMNs in modifying melanoma behavior. Although we evaluated the circulating levels of neutrophil-related mediators and NET biomarkers in melanoma patients, our results were obtained mainly in vitro and perhaps do not completely reflect the in vivo situation. Most results were obtained using CMs (soluble factors) derived from melanoma cell lines. Since PMNs can infiltrate the TME, it would have been interesting to evaluate the possible effects of direct interactions between PMNs and melanoma cell lines. Immunohistochemical investigations would be useful to evaluate whether tumor infiltrating neutrophils locally produce NETs in melanoma. Finally, a larger cohort of patients should be recruited to corroborate these data and to evaluate any differences in the circulating levels of neutrophil-related mediators and NETs among patients with melanoma at different stages of progression. Further studies are necessary to better understand the in vivo role of this bidirectional cross talk in patients and to evaluate the role of PMNs as therapeutic targets in human melanoma.

## Supplementary Information

Below is the link to the electronic supplementary material.

Supplementary file 1.

## Data Availability

The data presented in this study are available on request from the corresponding author.

## Abbreviations

CitH3: Citrullinated histone H3
CM: Conditioned medium
CTLA-4: Cytotoxic T lymphocyte-associated antigen 4
FMO: Fluorescence minus one
GM-CSF: Granulocyte–macrophage–colony-stimulating factor
ICIs: Immune checkpoint inhibitors
MFI: Median fluorescence intensity
MPO: Myeloperoxidase
MMP-9: Matrix metalloproteinase 9
NETs: Neutrophil extracellular traps
OS: Overall survival
PD-1: Programmed cell death protein 1
PDL-1: Programmed cell death protein 1 ligand
PI: Propidium iodide
PMNs: Polymorphonuclear neutrophils
TME: Tumor microenvironment
VEGF: Vascular endothelial growth factor

## References

[1] Miller KD, Nogueira L, Devasia T, Mariotto AB, Yabroff KR, Jemal A, Kramer J, Siegel RL (2022). Cancer treatment and survivorship statistics, 2022. CA Cancer J Clin.

[2] Forsea AM (2020). Melanoma epidemiology and early detection in Europe: diversity and disparities. Dermatol Pract Concept.

[3] Hodi FS, Sileni VC, Lewis KD (2022). Long-term survival in advanced melanoma for patients treated with nivolumab plus ipilimumab in CheckMate 067. J Clin Oncol.

[4] Wolchok JD, Chiarion-Sileni V, Gonzalez R (2022). Long-term outcomes with nivolumab plus ipilimumab or nivolumab alone versus ipilimumab in patients with advanced melanoma. J Clin Oncol.

[5] Ribas A, Hu-Lieskovan S (2016). What does PD-L1 positive or negative mean?. J Exp Med.

[6] Ramos RN, de Moraes CJ, Zelante B, Barbuto JA (2013). What are the molecules involved in regulatory T-cells induction by dendritic cells in cancer?. Clin Dev Immunol.

[7] Hartley GP, Chow L, Ammons DT, Wheat WH, Dow SW (2018). Programmed cell death ligand 1 (PD-L1) signaling regulates macrophage proliferation and activation. Cancer Immunol Res.

[8] Luo Q, Huang Z, Ye J (2016). PD-L1-expressing neutrophils as a novel indicator to assess disease activity and severity of systemic lupus erythematosus. Arthritis Res Ther.

[9] Buddhisa S, Rinchai D, Ato M, Bancroft GJ, Lertmemongkolchai G (2015). Programmed death ligand 1 on Burkholderia pseudomallei-infected human polymorphonuclear neutrophils impairs T cell functions. J Immunol.

[10] McNab FW, Berry MP, Graham CM (2011). Programmed death ligand 1 is over-expressed by neutrophils in the blood of patients with active tuberculosis. Eur J Immunol.

[11] Cristinziano L, Modestino L, Capone M (2022). PD-L1(+) neutrophils as novel biomarkers for stage IV melanoma patients treated with nivolumab. Front Immunol.

[12] Galdiero MR, Varricchi G, Loffredo S, Mantovani A, Marone G (2017). Roles of neutrophils in cancer growth and progression. J Leukoc Biol.

[13] Mantovani A, Cassatella MA, Costantini C, Jaillon S (2011). Neutrophils in the activation and regulation of innate and adaptive immunity. Nat Rev Immunol.

[14] Donskov F (2013). Immunomonitoring and prognostic relevance of neutrophils in clinical trials. Semin Cancer Biol.

[15] Galdiero MR, Varricchi G, Loffredo S (2018). Potential involvement of neutrophils in human thyroid cancer. PLoS ONE.

[16] Bald T, Quast T, Landsberg J (2014). Ultraviolet-radiation-induced inflammation promotes angiotropism and metastasis in melanoma. Nature.

[17] Rotondo R, Bertolotto M, Barisione G (2011). Exocytosis of azurophil and arginase 1-containing granules by activated polymorphonuclear neutrophils is required to inhibit T lymphocyte proliferation. J Leukoc Biol.

[18] Rawat K, Syeda S, Shrivastava A (2022). Hyperactive neutrophils infiltrate vital organs of tumor bearing host and contribute to gradual systemic deterioration via upregulated NE, MPO and MMP-9 activity. Immunol Lett.

[19] Rawat K, Syeda S, Shrivastava A (2021). Neutrophil-derived granule cargoes: paving the way for tumor growth and progression. Cancer Metastasis Rev.

[20] Brinkmann V (2018). Neutrophil extracellular traps in the second decade. J Innate Immun.

[21] Cristinziano L, Modestino L, Antonelli A, Marone G, Simon HU, Varricchi G, Galdiero MR (2022). Neutrophil extracellular traps in cancer. Semin Cancer Biol.

[22] Blenman KRM, Wang J, Cowper S, Bosenberg M (2019). Pathology of spontaneous and immunotherapy-induced tumor regression in a murine model of melanoma. Pigment Cell Melanoma Res.

[23] Schedel F, Mayer-Hain S, Pappelbaum KI (2020). Evidence and impact of neutrophil extracellular traps in malignant melanoma. Pigment Cell Melanoma Res.

[24] Muzio M, Re F, Sironi M, Polentarutti N, Minty A, Caput D, Ferrara P, Mantovani A, Colotta F (1994). Interleukin-13 induces the production of interleukin-1 receptor antagonist (IL-1ra) and the expression of the mRNA for the intracellular (keratinocyte) form of IL-1ra in human myelomonocytic cells. Blood.

[25] Calzetti F, Tamassia N, Arruda-Silva F, Gasperini S, Cassatella MA (2017). The importance of being "pure" neutrophils. J Allergy Clin Immunol.

[26] Borriello F, Iannone R, Di Somma S (2016). GM-CSF and IL-3 modulate human monocyte TNF-alpha production and renewal in in vitro models of trained immunity. Front Immunol.

[27] Dhawan P, Richmond A (2002). Role of CXCL1 in tumorigenesis of melanoma. J Leukoc Biol.

[28] Waugh DJ, Wilson C (2008). The interleukin-8 pathway in cancer. Clin Cancer Res.

[29] Barreda DR, Hanington PC, Belosevic M (2004). Regulation of myeloid development and function by colony stimulating factors. Dev Comp Immunol.

[30] Borriello F, Iannone R, Di Somma S (2017). Lipopolysaccharide-elicited TSLPR expression enriches a functionally discrete subset of human CD14(+) CD1c(+) monocytes. J Immunol.

[31] Kutsuna H, Suzuki K, Kamata N, Kato T, Hato F, Mizuno K, Kobayashi H, Ishii M, Kitagawa S (2004). Actin reorganization and morphological changes in human neutrophils stimulated by TNF, GM-CSF, and G-CSF: the role of MAP kinases. Am J Physiol Cell Physiol.

[32] Sabatini M, Chavez J, Mundy GR, Bonewald LF (1990). Stimulation of tumor necrosis factor release from monocytic cells by the A375 human melanoma via granulocyte-macrophage colony-stimulating factor. Cancer Res.

[33] Yousefi S, Mihalache C, Kozlowski E, Schmid I, Simon HU (2009). Viable neutrophils release mitochondrial DNA to form neutrophil extracellular traps. Cell Death Differ.

[34] Alfaro C, Teijeira A, Onate C (2016). Tumor-produced interleukin-8 attracts human myeloid-derived suppressor cells and elicits extrusion of neutrophil extracellular traps (NETs). Clin Cancer Res.

[35] Ferrucci PF, Gandini S, Battaglia A (2015). Baseline neutrophil-to-lymphocyte ratio is associated with outcome of ipilimumab-treated metastatic melanoma patients. Br J Cancer.

[36] Capone M, Giannarelli D, Mallardo D (2018). Baseline neutrophil-to-lymphocyte ratio (NLR) and derived NLR could predict overall survival in patients with advanced melanoma treated with nivolumab. J Immunother Cancer.

[37] Scapini P, Marini O, Tecchio C, Cassatella MA (2016). Human neutrophils in the saga of cellular heterogeneity: insights and open questions. Immunol Rev.

[38] Pal HC, Baxter RD, Hunt KM, Agarwal J, Elmets CA, Athar M, Afaq F (2015). Fisetin, a phytochemical, potentiates sorafenib-induced apoptosis and abrogates tumor growth in athymic nude mice implanted with BRAF-mutated melanoma cells. Oncotarget.

[39] Rossi S, Cordella M, Tabolacci C (2018). TNF-alpha and metalloproteases as key players in melanoma cells aggressiveness. J Exp Clin Cancer Res.

[40] Navarini-Meury AA, Conrad C (2009). Melanoma and innate immunity—active inflammation or just erroneous attraction? Melanoma as the source of leukocyte-attracting chemokines. Semin Cancer Biol.

[41] Zhou X, Peng M, He Y, Peng J, Zhang X, Wang C, Xia X, Song W (2021). CXC chemokines as therapeutic targets and prognostic biomarkers in skin cutaneous melanoma microenvironment. Front Oncol.

[42] Erdrich J, Lourdault K, Judd A, Kaufman D, Gong KW, Gainsbury M, Deng N, Shon W, Essner R (2022). Four immune modulating genes in primary melanoma that predict metastatic potential. J Surg Res.

[43] Filimon A, Preda IA, Boloca AF, Negroiu G (2021). Interleukin-8 in melanoma pathogenesis, prognosis and therapy: an integrated view into other neoplasms and chemokine networks. Cells.

[44] Nozawa H, Chiu C, Hanahan D (2006). Infiltrating neutrophils mediate the initial angiogenic switch in a mouse model of multistage carcinogenesis. Proc Natl Acad Sci USA.

[45] Fu X, Kassim SY, Parks WC, Heinecke JW (2001). Hypochlorous acid oxygenates the cysteine switch domain of pro-matrilysin (MMP-7). A mechanism for matrix metalloproteinase activation and atherosclerotic plaque rupture by myeloperoxidase. J Biol Chem.

[46] Valadez-Cosmes P, Raftopoulou S, Mihalic ZN, Marsche G, Kargl J (2022). Myeloperoxidase: growing importance in cancer pathogenesis and potential drug target. Pharmacol Ther.

[47] Hendrix AY, Kheradmand F (2017). The role of matrix metalloproteinases in development, repair, and destruction of the lungs. Prog Mol Biol Transl Sci.

[48] Cristinziano L, Modestino L, Loffredo S (2020). Anaplastic thyroid cancer cells induce the release of mitochondrial extracellular DNA traps by viable neutrophils. J Immunol.

[49] Demers M, Wong SL, Martinod K, Gallant M, Cabral JE, Wang Y, Wagner DD (2016). Priming of neutrophils toward NETosis promotes tumor growth. Oncoimmunology.

[50] Yousefi S, Simon HU (2016). NETosis: does it really represent nature's "suicide bomber"?. Front Immunol.

[51] Gupta S, Chan DW, Zaal KJ, Kaplan MJ (2018). A high-throughput real-time imaging technique to quantify NETosis and distinguish mechanisms of cell death in human neutrophils. J Immunol.

[52] Brinkmann V, Goosmann C, Kuhn LI, Zychlinsky A (2012). Automatic quantification of in vitro NET formation. Front Immunol.

[53] Kraaij T, Tengstrom FC, Kamerling SW, Pusey CD, Scherer HU, Toes RE, Rabelink TJ, van Kooten C, Teng YK (2016). A novel method for high-throughput detection and quantification of neutrophil extracellular traps reveals ROS-independent NET release with immune complexes. Autoimmun Rev.

[54] Xiao F, Jiang Y, Wang X, Jiang W, Wang L, Zhuang X, Zheng C, Ni Y, Chen L (2018). NETosis may play a role in the pathogenesis of Hashimoto's thyroiditis. Int J Clin Exp Pathol.

[55] Nikkola J, Vihinen P, Vuoristo MS, Kellokumpu-Lehtinen P, Kahari VM, Pyrhonen S (2005). High serum levels of matrix metalloproteinase-9 and matrix metalloproteinase-1 are associated with rapid progression in patients with metastatic melanoma. Clin Cancer Res.

[56] Mancuso F, Lage S, Rasero J (2020). Serum markers improve current prediction of metastasis development in early-stage melanoma patients: a machine learning-based study. Mol Oncol.

[57] Yousefi S, Simon D, Stojkov D, Karsonova A, Karaulov A, Simon HU (2020). In vivo evidence for extracellular DNA trap formation. Cell Death Dis.

[58] Grilz E, Mauracher LM, Posch F, Konigsbrugge O, Zochbauer-Muller S, Marosi C, Lang I, Pabinger I, Ay C (2019). Citrullinated histone H3, a biomarker for neutrophil extracellular trap formation, predicts the risk of mortality in patients with cancer. Br J Haematol.

