# Supplementary material for: Melanoma-derived soluble mediators modulate neutrophil biological properties and the release of neutrophil extracellular traps
Source: Cancer Immunol Immunother. 2023 Jul 31;72(10):3363–76. doi: 10.1007/s00262-023-03493-5 (PMC10491523; doi:10.1007/s00262-023-03493-5)
Supplement: Supplementary file 1 [file 262_2023_3493_MOESM1_ESM.pdf]

Supplementary Figure 1

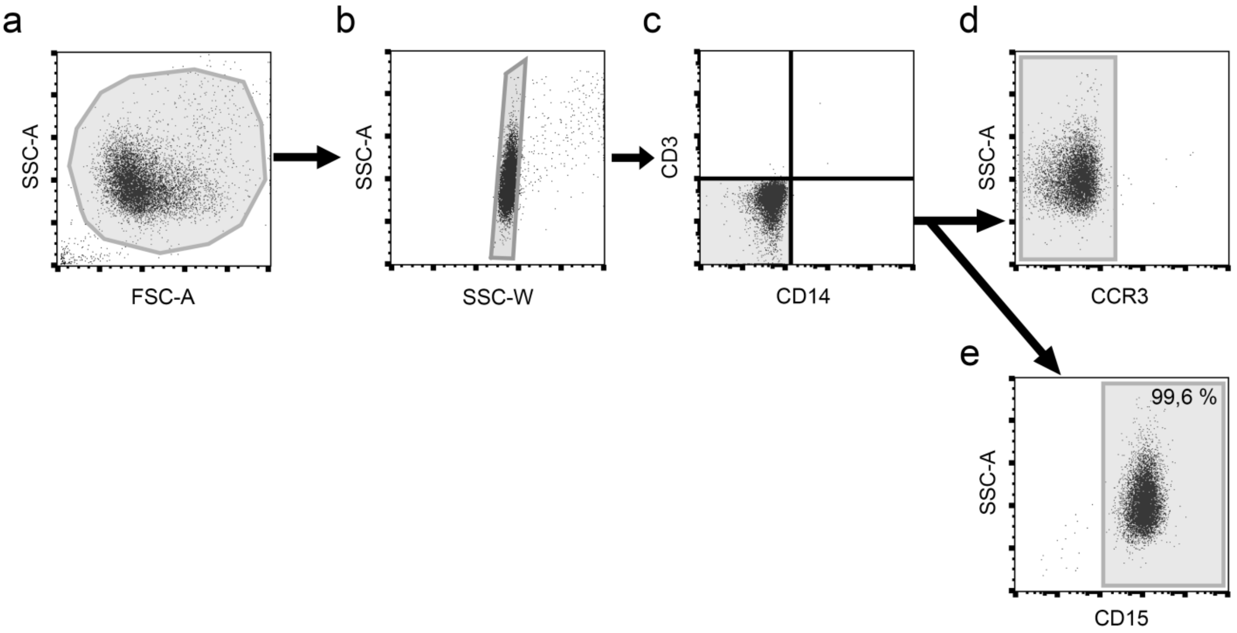

Supplementary Figure 1. Phenotipical analysis of purified human polymorphonuclear neutrophils (PMNs). PMNs were purified from peripheral blood of healthy donors and stained with the monoclonal antibodies as indicated. Flow cytometry plots were gated on live cells and show forward (FSC) and side scatter (SSC) of EasySep-purified untouched neutrophils (**a, b**). PMNs were negative for CD3, CD14 and CCR3 (**c, d**), and positive for CD15 (**e**).

Supplementary Table 1. A375-derived CM promoted neutrophil survival.

	Mean ± SEM			
	**CTRL**	**HEMa CM**	**SKMEL28 CM**	**A375 CM**
Live Cells	50.97 ± 3.69	38.04 ± 2.68	45.73 ± 3.45	74.45 ± 4.51 ***;**;#**
Apoptotic Cells	47.24 ± 3.85 **§**	59.91 ± 3.08 **§**	46.19 ± 3.02 **§**	20.48 ± 4.44
Necrotic Cells	1.79 ± 0.29	2.06 ± 0.61	8.06 ± 1.97	5.05 ± 0.53

	Mean ± SEM			
	**CTRL ISO**	**CTRL αGM-CSF**	**A375 CM ISO**	**A375 CM αGM-CSF**
Live Cells	50.12 ± 3.16	41.49 ± 3.13	64.71 ± 2.11 **£.** °	47.78 ± 5.65 **&**
Apoptotic Cells	45.62 ± 2.44 **$**	53.79 ± 3.47 **$**	25.48 ± 3.56	45.33 ± 6.15 **$**
Necrotic Cells	4.25 ± 1.03	4.74 ± 1.33	9.82 ± 2.11	8.40 ± 2.21

Polymorphonuclear neutrophils (PMNs) were cultured in melanoma-CM (SKMEL28 CM, A375 CM), HEMa CM, or control medium for 24 h. PMNs were then stained with FITC-conjugated annexin V and propidium iodide (PI) and evaluated by flow cytometry. Results are expressed as percentages of live, apoptotic or necrotic cells (mean ± SEM of five independent experiments); One-Way Anova and Dunn's multiple comparison test. PMN survival in A375-derived CM was evaluated in the presence of an anti-GM-CSF blocking antibody or a relative isotype control (10 µg/mL). At 24 h, live cells were stained with FITC-conjugated annexin V and propidium iodide (PI) and analyzed by flow cytometry. Results are expressed as percentages of live, apoptotic or necrotic cells (mean ± SEM of five independent experiments). One-Way Anova and Dunn's multiple comparison test.

***** p<0.05 versus CTRL; ****** versus HEMa CM; **#** versus SKMEL28 CM; **§** versus A375 CM; **£** versus CTRL ISO; ° versus CTRL αGM-CSF; **&** versus A375 CM ISO;**$** versus A375 ISO
